# Measuring and imaging of transcutaneous bilirubin, hemoglobin, and melanin based on diffuse reflectance spectroscopy

**DOI:** 10.1117/1.JBO.28.10.107001

**Published:** 2023-10-31

**Authors:** Masafumi Minakawa, Md. Abdul Wares, Kazuya Nakano, Hideaki Haneishi, Yoshihisa Aizu, Yoshio Hayasaki, Tetsuo Ikeda, Hajime Nagahara, Izumi Nishidate

**Affiliations:** a Tokyo University of Agriculture and Technology, Graduate School of Bio-Applications and Systems Engineering, Tokyo, Japan; b Government of Bangladesh, Ministry of Fisheries and Livestock, Department of Livestock Services, Dhaka, Bangladesh; c Seikei University, Department of Science and Technology, Faculty of Science and Technology, Tokyo, Japan; d Chiba University, Center for Frontier Medical Engineering, Chiba, Japan; e Muroran Institute of Technology, College of Design and Manufacturing Technology, Hokkaido, Japan; f Utsunomiya University, Center for Optical Research and Education, Tochigi, Japan; g Fukuoka Dental College, Section of General Surgery, Division of Oral and Medical Management, Department of Medicine, Fukuoka, Japan; h Osaka University, Institute for Datability Science, Osaka, Japan

**Keywords:** diffuse reflectance spectroscopy, Monte Carlo simulation, multiple regression analysis, hemoglobin, tissue oxygen saturation, bilirubin, melanin, jaundice, melanogenesis

## Abstract

**Significance:**

Evaluation of biological chromophore levels is useful for detection of various skin diseases, including cancer, monitoring of health status and tissue metabolism, and assessment of clinical and physiological vascular functions. Clinically, it is useful to assess multiple different chromophores *in vivo* with a single technique or instrument.

**Aim:**

To investigate the possibility of estimating the concentration of four chromophores, bilirubin, oxygenated hemoglobin, deoxygenated hemoglobin, and melanin from diffuse reflectance spectra in the visible region.

**Approach:**

A new diffuse reflectance spectroscopic method based on the multiple regression analysis aided by Monte Carlo simulations for light transport was developed to quantify bilirubin, oxygenated hemoglobin, deoxygenated hemoglobin, and melanin. Three different experimental animal models were used to induce hyperbilirubinemia, hypoxemia, and melanogenesis in rats.

**Results:**

The estimated bilirubin concentration increased after ligation of the bile duct and reached around 18  mg/dl at 50 h after the onset of ligation, which corresponds to the reference value of bilirubin measured by a commercially available transcutaneous bilirubin meter. The concentration of oxygenated hemoglobin and that of deoxygenated hemoglobin decreased and increased, respectively, as the fraction of inspired oxygen decreased. Consequently, the tissue oxygen saturation dramatically decreased. The time course of melanin concentration after depilation of skin on the back of rats was indicative of the supply of melanosomes produced by melanocytes of hair follicles to the growing hair shaft.

**Conclusions:**

The results of our study showed that the proposed method is capable of the *in vivo* evaluation of percutaneous bilirubin level, skin hemodynamics, and melanogenesis in rats, and that it has potential as a tool for the diagnosis and management of hyperbilirubinemia, hypoxemia, and pigmented skin lesions.

## 1 Introduction

Evaluation of biological chromophore levels is useful for detection of various skin diseases, including cancer, monitoring of health status and tissue metabolism, and assessment of clinical and physiological vascular functions. The major chromophores in the superficial layer of skin are oxygenated hemoglobin, deoxygenated hemoglobin, and melanin, which have distinct optical absorption properties in the visible wavelength range. Hemoglobin has two main derivatives, oxygenated and deoxygenated hemoglobin. The absorption spectra of oxygenated and deoxygenated hemoglobin are different. This is because the binding of oxygen to hemoglobin changes the light absorption spectrum of hemoglobin.^1^ Varying the concentrations of oxygenated and deoxygenated hemoglobin will alter the diffuse reflectance spectrum. A continuous supply of oxygen is required for peripheral skin tissues and cells. Oxygen is delivered through the bloodstream. The delivery of oxygen to the peripheral skin tissues can be evaluated from the diffuse reflectance spectrum, which is based on the absorption spectra of oxygenated and deoxygenated hemoglobin. The percentage of oxygenated hemoglobin to total hemoglobin in a volume of tissue is referred to as tissue oxygen saturation (${\mathrm{StO}}_{2}$) or hemoglobin oxygen saturation.^2^ It is a useful indicator to monitor peripheral tissue oxygen consumption, hypoperfusion, and cyanosis.

Melanin is synthesized at ∼10  nm granule sites that are scattered along the inner wall of melanosomes, organelles that are ∼1  μm in diameter. Melanin is the major chromophore of human skin with several potential biological functions, including protection from solar radiation and antioxidant defense.^3^^,^^4^ The absorption of light by the epidermis is usually dominated by the absorption of light by melanin in the majority of individuals. Melanin has a broad absorption spectrum, with stronger absorption at shorter wavelengths in the range from the ultraviolet to the near infrared.^5^^–^^7^ Melanin has also been implicated in pigmented skin lesions such as freckles, lentigines, melisma, seborrheic keratosis, solar keratosis, basal cell carcinoma, and melanoma. Therefore, estimating the amount of melanin in the skin is important for diagnosis of benign and malignant pigmented skin lesions. Melanin in the epidermis is a superficial absorber that can greatly affect the penetration of light into the epidermis and dermis layers of skin tissue. The color of human skin is dependent on the amount of melanin in the epidermis. Therefore, evaluation of the amount of melanin in the skin is also important for dosimetry in light therapy, such as photodynamic therapy.

Bilirubin is also an important chromophore for evaluating health conditions such as neonatal jaundice, liver cirrhosis, and hepatitis. Bilirubin is a hemoglobin breakdown product and has a broad absorption spectrum with a maximum absorption in the range of 400 to 500 nm. A condition in which the serum bilirubin concentration exceeds the holding capacity of the serum albumin is known as hyperbilirubinemia or jaundice. This condition is responsible for the yellowish skin color in jaundice. In most infants, hyperbilirubinemia reflects a normal transitional phenomenon called physiological jaundice. In some infants, however, significant hyperbilirubinemia may cause bilirubin to accumulate in the brain tissue, potentially causing irreversible brain damage called kernicterus.^8^ It is therefore recommended to carefully monitor serum bilirubin or transcutaneous bilirubin^9^^,^^10^ levels in neonate jaundice, especially in the first 24 h.

When the concentration of each chromophore varies, the corresponding change may be observed on diffusely reflected light from the skin tissue in the visible wavelength range. Steady-state diffuse reflectance spectra with a continuous-wave light can be easily acquired using a white light source, inexpensive optical components, and a spectrometer. Therefore, analysis of diffuse reflectance spectra may provide useful information on tissue activities and functions that are related to melanin and hemoglobin. Diffuse reflectance spectroscopy has been widely used for the evaluation of human skin chromophores at a single region.^11^^–^^19^

Palmer and Ramanujam^20^ have developed a method for the extraction of absorption and scattering coefficients from the spectral diffuse reflectance measured by any arbitrary probe geometry, which is based on an inverse Monte Carlo modeling of the light transport. They also applied the method to a set of diffuse reflectance spectra of breast tissue and demonstrated the ability to classify a sample as malignant or non-malignant with a cross-validated sensitivity and specificity of 82% and 92%, respectively.^21^ To assess the robustness and clinical utility of the algorithm developed by Palmer et al., Bender et al.^22^ demonstrated the quantitative accuracy of the extraction of optical properties from diffuse reflectance spectra of tissue mimicking phantoms under different instruments and fiber optic probes. Hennessy et al.^23^ proposed a method that uses a look-up table based on Monte Carlo simulation (MCS) to extract both the reduced scattering coefficient and the absorption coefficient from diffuse reflectance spectra and to estimate the hemoglobin concentration. They demonstrated the good performance of the method using tissue-mimicking phantoms. The error rates were 1.74%, 0.74%, and 2.42% for the reduced scattering coefficient, absorption coefficients, and hemoglobin concentrations, respectively.

Randeberg et al.^24^ reported the algorithms based on diffusion theory for the estimation of a transcutaneous bilirubin index (TcB) from the measurement of the diffuse reflectance spectrum. They showed that the estimated TcB correlated well with total serum bilirubin (r=0.81, p<0.05). Using MCS for light transport, Delgado Atencio et al.^25^ numerically studied the diffuse reflectance spectra of neonatal skin with bilirubin concentrations ranging from physiological to toxic, and evaluated the influence of pigmentation and blood content on the spectra.

Multi-spectral imaging^26^^–^^28^ and hyperspectral imaging^29^^–^^32^ based on diffuse reflectance spectroscopy have been widely employed for evaluating the spatial distribution of chromophore contents in living tissue. A simple method for quantitative measurements and imaging of melanin and hemoglobin concentrations in *in vivo* skin tissue based on diffuse reflectance images at 6 wavelengths (500, 520, 540, 560, 580, and 600 nm) using multiple regression analysis (MRA) aided by MCSs has been previously proposed.^16^^,^^33^ In the above studies, two or three of the four chromophores (oxygenated hemoglobin, deoxygenated hemoglobin, melanin, bilirubin) have been evaluated. However, there is no study that targets all four chromophores. Some of the other methods described in the studies presented above seem to be potentially capable of being extended to estimate all four chromophores. Nevertheless, they did not perform an estimation of the all four chromophores. We extend the method previously proposed^16^^,^^33^ to the quantification of oxygenated hemoglobin, deoxygenated hemoglobin, melanin, and bilirubin.

The aim of the present study is to investigate the possibility of estimating the concentration of four chromophores, bilirubin ($C_{\mathrm{bil}}$), oxygenated hemoglobin ($C_{\mathrm{oh}}$), deoxygenated hemoglobin ($C_{\mathrm{dh}}$), and melanin ($C_{m}$) from diffuse reflectance spectra in the visible wavelength region. The proposed approach utilizes MRA aided by MCSs for diffuse reflectance spectra of skin tissue. Using the absorbance spectrum as a dependent variable and the extinction coefficients of bilirubin, melanin, oxygenated hemoglobin, and deoxygenated hemoglobin as independent variables, MRA provides regression coefficients. Concentrations of bilirubin, melanin, oxygenated hemoglobin, and deoxygenated hemoglobin are then determined from the regression coefficients using empirical formulae that are deduced numerically in advance.

To confirm the feasibility of this method for evaluating hyperbilirubinemia, hemodynamics, and melanogenesis in skin tissues, we performed *in vivo* experiments with rat dorsal skin. An experimental model of obstructive jaundice caused by bile duct ligation in rats was introduced to evaluate hyperbilirubinemia quantitatively. Experiments with rat dorsal skin while changing the fraction of inspired oxygen (${\mathrm{FiO}}_{2}$) were performed to demonstrate the ability of the method to estimate the total hemoglobin concentration and tissue oxygen saturation under the conditions of normoxia, hypoxia, and anoxia. In addition to the experiments with albino rats, we also investigated the concentration of melanin in the dorsal skin of pigmented Long-Evans rats after hair removal treatment.

## 2 Principle

**Fig. 1:**
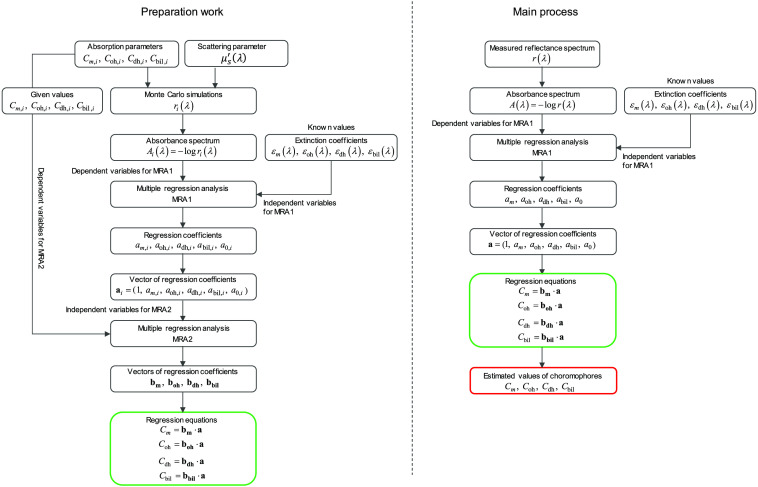
Flowchart of the process for estimating the concentrations of melanin $C_{m}$, oxygenated hemoglobin $C_{\mathrm{oh}}$, deoxygenated hemoglobin $C_{\mathrm{dh}}$, and bilirubin $C_{\mathrm{bil}}$. (a) Preparation work for determining the regression equations and (b) main process for deriving $C_{m}$, $C_{\mathrm{oh}}$, $C_{\mathrm{dh}}$, and $C_{\mathrm{bil}}$ from the measured reflectance spectra.

Figure 1 shows a flow diagram of the method used to estimate the concentrations of melanin, oxygenated hemoglobin, deoxygenated hemoglobin, and bilirubin. An attenuation spectrum A(λ) is defined as

$$A(\lambda )=-\log_{10}\;r(\lambda ),$$ (1)

where r(λ) is the diffuse reflectance spectrum expressed by the ratio between reflected light intensity spectrum measured on tissue $i_{t}(\lambda )$ and that on a standard white diffuser $i_{\mathrm{std}}(\lambda )$ as

$$r(\lambda )=\frac{i_{t}}{i_{\mathrm{std}}}.$$ (2)

We assumed that the skin tissue consists of epidermis containing melanin, and dermis containing oxygenated hemoglobin, deoxygenated hemoglobin, and bilirubin. The attenuation spectrum A(λ) can be approximated as the sum of attenuations due to chromophores in the skin as

$$A(\lambda )=C_{m}l_{e}(\lambda ,\mu_{s}^{\prime }(\lambda ),C_{m})\varepsilon_{m}(\lambda )+C_{\mathrm{oh}}l_{d}(\lambda ,\mu_{s}^{\prime }(\lambda ),C_{\mathrm{oh}},C_{\mathrm{dh}},C_{\mathrm{bil}})\varepsilon_{\mathrm{oh}}(\lambda )\;+C_{\mathrm{dh}}l_{d}(\lambda ,\mu_{s}^{\prime }(\lambda ),C_{\mathrm{oh}},C_{\mathrm{dh}},C_{\mathrm{bil}})\varepsilon_{\mathrm{dh}}(\lambda )+C_{\mathrm{bil}}l_{d}(\lambda ,\mu_{s}^{\prime }(\lambda ),C_{\mathrm{oh}},C_{\mathrm{dh}},C_{\mathrm{bil}})\varepsilon_{\mathrm{bil}}(\lambda )\;+D(\lambda ,\mu_{s}^{\prime }(\lambda ),\mu_{a,\text{baseline}}),$$ (3)

where C (M) is the molar concentration, l (cm) is the mean path length, $\mu_{s^{\prime }}(\lambda )$ (${\mathrm{cm}}^{-1}$) is the reduced scattering coefficient spectrum and ε(λ) (${\mathrm{cm}}^{-1}\;M^{-1}$) is the extinction coefficient. The subscripts m, oh, dh, and bil denote melanin, oxygenated hemoglobin, deoxygenated hemoglobin, and bilirubin, respectively. The subscripts e and d for mean path length l and the reduced scattering coefficient $\mu_{s^{\prime }}(\lambda )$ denote epidermis and dermis. Under conditions where absorption is comparable to or greater than scattering, the path length depends not only on scattering but also on absorption. Therefore, the effective path lengths are shorter in turbid media with strong absorption. This effect is significant at visible wavelengths in tissue, where there is strong absorption due to biological chromophores such as oxygenated hemoglobin and deoxygenated hemoglobin.^34^ Therefore, we consider the dependence of the path lengths to not only scattering but also absorption. D indicates attenuation due to scattering and baseline absorption associated with the skin tissue $\mu_{a,\text{baseline}}$ (λ) (${\mathrm{cm}}^{-1}$) that is free of melanin, hemoglobin, and bilirubin. The absorption coefficient of the skin tissue was assumed to depend on the concentrations of m, oh, dh, and bil $\left(C_{m},C_{\mathrm{oh}},C_{\mathrm{dh}},C_{\mathrm{bil}}\right)$ as

$$\mu_{a}(\lambda )=2.303(C_{m}\varepsilon_{m}(\lambda )+C_{\mathrm{oh}}\varepsilon_{\mathrm{oh}}(\lambda )+C_{\mathrm{dh}}\varepsilon_{\mathrm{dh}}(\lambda )+C_{\mathrm{bil}}\varepsilon_{\mathrm{bil}}(\lambda )).$$ (4)

The total hemoglobin concentration $C_{\mathrm{th}}$ is defined as the sum of $C_{\mathrm{oh}}$ and $C_{\mathrm{dh}}$ as follows:

$$C_{\mathrm{th}}=C_{\mathrm{oh}}+C_{\mathrm{dh}}.$$ (5)

The tissue oxygen saturation is determined as

$${\mathrm{StO}}_{2}=100\times (C_{\mathrm{oh}}/C_{\mathrm{th}}).$$ (6)

Using A(λ) as the response variable and ε(λ) as the predictor variables, MRA1 can be applied to Eq. (3) as

$$A(\lambda )=a_{m}\varepsilon_{m}(\lambda )+a_{\mathrm{oh}}\varepsilon_{\mathrm{oh}}(\lambda )+a_{\mathrm{dh}}\varepsilon_{\mathrm{dh}}(\lambda )+a_{\mathrm{bil}}\varepsilon_{\mathrm{bil}}(\lambda )+a_{0},$$ (7)

where $a_{m}$, $a_{\mathrm{oh}}$, $a_{\mathrm{dh}}$, $a_{\mathrm{bil}}$, and $a_{0}$ are the regression coefficients and are statistically calculated. The regression coefficients $a_{m}$, $a_{\mathrm{oh}}$, $a_{\mathrm{dh}}$, and $a_{\mathrm{bil}}$ describe the degree of contribution of each extinction coefficient to A(λ) and are related to the concentrations $C_{m}$, $C_{\mathrm{oh}}$, $C_{\mathrm{dh}}$, and $C_{\mathrm{bil}}$, respectively. The regression coefficient $a_{0}$ represents the bias component of A(λ) and mathematically determined in MRA as

$$a_{0}=\overset{¯}{A}-{\overset{¯}{\varepsilon }}_{m}a_{m}-{\overset{¯}{\varepsilon }}_{\mathrm{oh}}a_{\mathrm{oh}}-{\overset{¯}{\varepsilon }}_{\mathrm{dh}}a_{\mathrm{dh}}-{\overset{¯}{\varepsilon }}_{\mathrm{bil}}a_{\mathrm{bil}},$$ (8)

where $\overset{¯}{A}$, ${\overset{¯}{\varepsilon }}_{m}$, ${\overset{¯}{\varepsilon }}_{\mathrm{oh}}$, ${\overset{¯}{\varepsilon }}_{\mathrm{dh}}$, and ${\overset{¯}{\varepsilon }}_{\mathrm{bil}}$ are the averages of A(λ), $\varepsilon_{m}(\lambda )$, $\varepsilon_{\mathrm{oh}}(\lambda )$, $\varepsilon_{\mathrm{dh}}(\lambda )$, and $\varepsilon_{\mathrm{bil}}(\lambda )$ over the wavelength range, respectively. Thus, $a_{0}$ involves the degree of contribution of the attenuation due to each chromophore in the skin to the attenuation spectrum A(λ). The expression in Eq. (8) and the averages in it are derived from the principle of MRA.

To quantify the values of $C_{m}$, $C_{\mathrm{oh}}$, $C_{\mathrm{dh}}$, and $C_{\mathrm{bil}}$ using the regression coefficients of $a_{m}$, $a_{\mathrm{oh}}$, $a_{\mathrm{dh}}$, $a_{\mathrm{bil}}$, and $a_{0}$ obtained from MRA1, we consider the empirical formulae for $C_{m}$, $C_{\mathrm{oh}}$, $C_{\mathrm{dh}}$, and $C_{\mathrm{bil}}$ as

$$C_{m}=b_{m}\cdot a,$$ (9)

$$C_{\mathrm{oh}}=b_{\mathrm{oh}}\cdot a,$$ (10)

$$C_{\mathrm{dh}}=b_{\mathrm{dh}}\cdot a,$$ (11)

$$C_{\mathrm{bil}}=b_{\mathrm{bil}}\cdot a,$$ (12)

$$a={\left[1,a_{m},a_{\mathrm{oh}},a_{\mathrm{dh}},a_{\mathrm{bil}},a_{0}\right]}^{T},$$ (13)

$$b_{m}=[b_{m,0},b_{m,1},b_{m,2},b_{m,3},b_{m,4},b_{m,5}],$$ (14)

$$b_{\mathrm{oh}}=[b_{\mathrm{oh},0},b_{\mathrm{oh},1},b_{\mathrm{oh},2},b_{\mathrm{oh},3},b_{\mathrm{oh},4},b_{\mathrm{oh},5}],$$ (15)

$$b_{\mathrm{dh}}=[b_{\mathrm{dh},0},b_{\mathrm{dh},1},b_{\mathrm{dh},2},b_{\mathrm{dh},3},b_{\mathrm{dh},4},b_{\mathrm{dh},5}],$$ (16)

$$b_{\mathrm{bil}}=[b_{\mathrm{bil},0},b_{\mathrm{bil},1},b_{\mathrm{bil},2},b_{\mathrm{bil},3},b_{\mathrm{bil},4},b_{\mathrm{bil},5}],$$ (17)

where the symbol ^*T*^ represents the transposition of a vector. The conversion vectors of $b_{m}$, $b_{\mathrm{oh}}$, $b_{\mathrm{dh}}$, and $b_{\mathrm{bil}}$ consist of the coefficients $b_{m,i}$, $b_{\mathrm{oh},i}$, $b_{\mathrm{dh},i}$, and $b_{\mathrm{bil},i}$ (i=0, 1, 2, 3, 4, 5), respectively, which are unknown and must be determined before estimating $C_{m}$, $C_{\mathrm{oh}}$, $C_{\mathrm{dh}}$, and $C_{\mathrm{bil}}$.

To determine reliable values of $b_{m,i}$, $b_{\mathrm{oh},i}$, $b_{\mathrm{dh},i}$, and $b_{\mathrm{bil}}$, we conduct further multiple regression analyses. We refer to this analysis as MRA2. In this analysis, the given values of $C_{m}$, $C_{\mathrm{oh}}$, $C_{\mathrm{dh}}$, and $C_{\mathrm{bil}}$ in MCS were regarded as dependent variables, and the five regression coefficients $a_{m}$, $a_{\mathrm{oh}}$, $a_{\mathrm{dh}}$, $a_{\mathrm{bil}}$, and $a_{0}$ were regarded as independent variables to determine the regression equations for $C_{m}$, $C_{\mathrm{oh}}$, $C_{\mathrm{dh}}$, and $C_{\mathrm{bil}}$. To derive the datasets of chromophores and the regression coefficients for MRA2, we generated 1800 diffuse reflectance spectra of skin tissue model at λ=460 to 590 nm with a 10 nm step using the MCS under the various values of $C_{m}$, $C_{\mathrm{oh}}$, $C_{\mathrm{dh}}$, and $C_{\mathrm{bil}}$. In the wavelength range 460 to 590 nm, oxygenated hemoglobin and deoxygenated hemoglobin have isosbestic points at 570 and 585 nm. Reflectance at 560 nm is sensitive to the oxygen state of hemoglobin. Bilirubin has an absorption peak at 460 nm. In this wavelength range, the absorption spectrum of melanin does not have the easily distinguishable peaks that present in the absorption spectra of other chromophores, but is characterized by a gradual decrease with increasing wavelength. This feature contributes to the spectral characteristics in which the diffuse reflectance is low at the shorter wavelength and high at the longer wavelength. The fact that the absorption spectrum of melanin is different from that of other chromophores makes it easier to estimate melanin. Therefore, we chose the wavelength range 460 to 590 nm to use for the MRA. We used the MCS source code developed by Wang et al.^35^ The simulation model consisted of two layers representing the epidermis and dermis. In a single simulation of diffuse reflectance at each wavelength, 5,000,000 photons were randomly launched. The absorption coefficients of oxygenated hemoglobin $\mu_{a,\mathrm{oh}}(\lambda )$ (${\mathrm{cm}}^{-1}$), deoxygenated hemoglobin $\mu_{a,\mathrm{dh}}(\lambda )$ (${\mathrm{cm}}^{-1}$), bilirubin $\mu_{a,\mathrm{bil}}(\lambda )$ (${\mathrm{cm}}^{-1}$), and melanin $\mu_{a,m}(\lambda )$ (${\mathrm{cm}}^{-1}$) were obtained from the values of $\varepsilon_{\mathrm{oh}}(\lambda )$ (${\mathrm{cm}}^{-1}M^{-1}$),^36^ $\varepsilon_{\mathrm{dh}}(\lambda )$ (${\mathrm{cm}}^{-1}M^{-1}$),^36^ $\varepsilon_{\mathrm{bil}}(\lambda )$ (${\mathrm{cm}}^{-1}M^{-1}$),^37^ and $\varepsilon_{m}(\lambda )$ (${\mathrm{cm}}^{-1}M^{-1}$),^3^ as shown in Table 1. The value of $\mu_{a}(\lambda )$ (${\mathrm{cm}}^{-1}$) was derived from the product of the molar concentration C (M) and the molar extinction coefficient ε(λ) (${\mathrm{cm}}^{-1}M^{-1}$) as, $\mu_{a}(\lambda )=2.303C\varepsilon (\lambda )$. The absorption coefficient of the epidermis depends on the volume concentration of melanin in the epidermis $C_{m}$. We used the absorption coefficient of a melanosome given in the literature^39^ as the absorption coefficient of melanin $\mu_{a,m}(\lambda )$ for the MCS. This corresponds to the absorption coefficient of the epidermis for the case in which $C_{m}=100$ vol.% (i.e., 0.21 M). We subsequently derived the absorption coefficients of the epidermis for 10 lower concentrations of $C_{m}=1$ to 10 vol.% at 1 − vol.% intervals (i.e., $C_{m}=2.14\times 10^{-3}$ to $21.4\times 10^{-3}\;M$ at $2.14\times 10^{-3}-M$ intervals), by simply proportioning them to that of $C_{m}=100\%$, and the absorption coefficients were input for the epidermis layer in the MCS.

**Table 1:** Values in the visible wavelength range between 460 and 590 nm of: (1) the molar extinction coefficients $\varepsilon_{\mathrm{oh}}(\lambda )$, $\varepsilon_{\mathrm{dh}}(\lambda )$, $\varepsilon_{\mathrm{bil}}(\lambda )$, and $\varepsilon_{m}(\lambda )$ of oxygenated hemoglobin, deoxygenated hemoglobin, bilirubin, and melanin, respectively; and (2) the reduced scattering coefficients $\mu_{s^{\prime }}(\lambda )$ of epidermis and dermis. References of these values are also provided.

	$\varepsilon_{\mathrm{oh}}(\lambda )$ (${\mathrm{cm}}^{-1}M^{-1}$)	$\varepsilon_{\mathrm{dh}}(\lambda )$ (${\mathrm{cm}}^{-1}M^{-1}$)	$\varepsilon_{\mathrm{bil}}(\lambda )$ (${\mathrm{cm}}^{-1}M^{-1}$)	$\varepsilon_{m}(\lambda )$ (${\mathrm{cm}}^{-1}M^{-1}$)	$\mu_{s^{\prime }}(\lambda )$ (${\mathrm{cm}}^{-1}$)
Wavelength (nm)	Ref. 36	Ref. 36	Ref. 37	Ref. 3	Ref. 38
460	896.52077	240.192	126.29952	52924.25352	64.94
470	834.56097	179.32968	87.24456	44859.32394	60.61
480	778.05557	143.79768	78.57	29928.05634	56.69
490	726.42535	127.89576	90.0936	14964.80282	53.13
500	679.16269	113.03712	112.6548	5564.21127	49.88
510	635.82156	108.19008	139.17744	1586.14085	46.93
520	596.00898	130.69296	170.58384	429.1831	44.22
530	559.37788	215.76672	210.79656	145.14085	41.74
540	525.62101	287.4744	251.5968	77.90141	39.46
550	494.46571	232.2864	288.4248	56.32394	37.36
560	465.66951	176.11128	290.4552	45.35992	35.43
570	439.01632	240.2784	243.3888	38.72665	33.64
580	414.31315	270.5616	199.908	34.71357	31.99
590	391.38731	77.76432	152.95176	32.28657	30.46

We assumed that the whole blood with $2.32\times 10^{-3}\;M$ of hemoglobin is 100% volume concentration of total hemoglobin ($C_{\mathrm{th}}=100$ vol.%) that is equivalent to 44% hematocrit and 150  g/L of hemoglobin. The sum of the absorption coefficients of oxygenated hemoglobin $\mu_{a,\mathrm{oh}}(\lambda )$ and deoxygenated hemoglobin $\mu_{a,\mathrm{dh}}(\lambda )$ represents the absorption coefficients of total hemoglobin $\mu_{a,\mathrm{th}}(\lambda )$ (${\mathrm{cm}}^{-1}$). The absorption coefficients for total hemoglobin $\mu_{a,\mathrm{th}}(\lambda )$ for the values of $C_{\mathrm{th}}=0.2$ to 1.0 vol.% at 0.2 − vol.% intervals (i.e., $C_{\mathrm{th}}=4.65\times 10^{-7}$ to $23.3\times 10^{-7}\;M$ at $4.65\times 10^{-7}-M$ intervals) were input for the dermis layer in the MCS. Tissue oxygen saturation (${\mathrm{StO}}_{2}$) was determined by $\mu_{a,\mathrm{oh}}(\lambda )/\mu_{a,\mathrm{th}}(\lambda )$, respectively, and values ranged from 0% to 100% were used for the simulation. The absorption coefficient of bilirubin $\mu_{a,\text{bil}}(\lambda )$ was derived as $\mu_{a,\text{bil}}(\lambda )=2.303(C_{\mathrm{bil}}/P_{\mathrm{Mbil}})\varepsilon_{\mathrm{bil}}(\lambda )$, where $C_{\mathrm{bil}}$ (g/L), $\varepsilon_{\mathrm{bil}}(\lambda )$ (${\mathrm{cm}}^{-1}M^{-1}$), and $P_{M\mathrm{bil}}$ (g/mol) are the bilirubin concentration in the whole blood, the extinction coefficient of bilirubin, and the gram molecular weight of bilirubin, respectively. The absorption coefficient of bilirubin was then scaled by a coefficient $f_{\mathrm{bil}}$. The value of $f_{\mathrm{bil}}$ was kept at 0.2.^40^ The values of $\mu_{a,\mathrm{bil}}(\lambda )$ for $C_{\mathrm{bil}}=0$, 1, 5, 10, 15, and 20  mg/dl (i.e., $C_{\mathrm{bil}}=0,0.17\times 10^{-4}$, $0.87\times 10^{-4}$, $1.71\times 10^{-4}$, $2.61\times 10^{-4}$, and $34.8\times 10^{-4}\;M$) were used as input for the dermis layer in the MCS. For all simulations, the refractive index of the epidermis and dermis layers was assumed to be the same and fixed at 1.4. The thicknesses of the epidermis and dermis layers were set to 0.06 and 4.94 mm, respectively. The thickness of the epidermis and dermis varies depending on the animal species and body part. We chose 0.06 mm for the epidermal thickness, which is the thickness assumed in the literature.^38^ It has been reported that the total thickness of the epidermis and dermis in human ranges from 1 to 4 mm.^41^ We set the total thickness of the epidermis and dermis to 5 mm to cover that range.

The reduced scattering coefficient $\mu_{s^{\prime }}(\lambda )$^38^ was used for the epidermis and dermis. For a proper simulation, different values of $\mu_{s^{\prime }}(\lambda )$ should be used for the epidermis and dermis. However, the values of the epidermis were not available in the literature for the analyzed wavelength range. Thus, the same values were used for both epidermis and dermis. Although there is surely some difference in $\mu_{s^{\prime }}(\lambda )$ between epidermis and dermis, the differences are not large.^38^ Moreover, thinness of the epidermis makes the details of light scattering of minor importance for visible wavelength applications involving photon diffusion. Also, subtle differences in light scattering are important for devices and techniques, which are primarily based on single scattering from the epidermis such as elastic backscatter, coherence backscatter, or polarized backscatter (Table 2).^38^

**Table 2:** Ranges of melanin concentration $C_{m}$, bilirubin concentration $C_{\mathrm{bil}}$, total hemoglobin concentration $C_{\mathrm{th}}$, and tissue oxygen saturation ${\mathrm{StO}}_{2}$ given to the MCS skin model.

	Unit	Epidermis	Dermis
$C_{m}$	vol.%	1, 2, 3, 4, 5, 6, 7, 8, 9, 10	—
	M	$2.14\times 10^{-3}$, $4.29\times 10^{-3}$, $6.43\times 10^{-3}$, $8.57\times 10^{-3}$, $10.7\times 10^{-3}$, $12.9\times 10^{-3}$, $15.0\times 10^{-3}$, $17.1\times 10^{-3}$, $19.3\times 10^{-3}$, $21.4\times 10^{-3}$	—
$C_{\mathrm{bil}}$	mg/dl	—	0, 1, 5, 10, 15, 20
	M	—	0, $0.17\times 10^{-4}$, $0.87\times 10^{-4}$, $1.71\times 10^{-4}$, $2.61\times 10^{-4}$, $34.8\times 10^{-4}$
$C_{\mathrm{th}}$	vol.%	—	0.2, 0.4, 0.6, 0.8, 1.0
	M		$4.65\times 10^{-7}$, $9.3\times 10^{-7}$, $14.0\times 10^{-7}$, $18.6\times 10^{-7}$, $23.3\times 10^{-7}$
${\mathrm{StO}}_{2}$	%	—	0, 20, 40, 60, 80, 100

The MRA1 analysis for each simulated spectrum based on Eq. (7) generated 1800 sets of vector a and concentrations $C_{m}$, $C_{\mathrm{oh}}$, $C_{\mathrm{dh}}$, and $C_{\mathrm{bil}}$. The conversion vectors $b_{m}$, $b_{\mathrm{oh}}$, $b_{\mathrm{dh}}$, and $b_{\mathrm{bil}}$ were determined statistically by performing MRA2. The extinction coefficients spectra of $\varepsilon_{\mathrm{oh}}(\lambda )$, $\varepsilon_{\mathrm{dh}}(\lambda )$, $\varepsilon_{\mathrm{bil}}(\lambda )$, and $\varepsilon_{m}(\lambda )$ are used in both preparation work and main process. Reflectance spectra used in the preparation work are simulated spectra generated by MCS while those used in main process are actually measured spectra. Conversion vectors obtained in the preparation work are used in the main process. Once $b_{m}$, $b_{\mathrm{oh}}$, $b_{\mathrm{dh}}$, and $b_{\mathrm{bil}}$ were obtained, $C_{m}$, $C_{\mathrm{oh}}$, $C_{\mathrm{dh}}$, and $C_{\mathrm{bil}}$ were calculated from $a_{m}$, $a_{\mathrm{oh}}$, $a_{\mathrm{dh}}$, $a_{\mathrm{bil}}$, and $a_{0}$, which were derived from MRA1 for the measured attenuation spectra A(λ), without the MCS, as shown in Fig. 1(b). All of these 1800 sets of vectors a and concentrations $C_{m}$, $C_{\mathrm{oh}}$, $C_{\mathrm{dh}}$, and $C_{\mathrm{bil}}$ were calculated only once. The conversion vectors $b_{m}$, $b_{\mathrm{oh}}$, $b_{\mathrm{dh}}$, and $b_{\mathrm{bil}}$ were also calculated only once. The conversion vectors $b_{m}$, $b_{\mathrm{oh}}$, $b_{\mathrm{dh}}$, and $b_{\mathrm{bil}}$ were determined statistically by performing MRA2. Once $b_{m}$, $b_{\mathrm{oh}}$, $b_{\mathrm{dh}}$, and $b_{\mathrm{bil}}$ were obtained, $C_{m}$, $C_{\mathrm{oh}}$, $C_{\mathrm{dh}}$, and $C_{\mathrm{bil}}$ were calculated from $a_{m}$, $a_{\mathrm{oh}}$, $a_{\mathrm{dh}}$, $a_{\mathrm{bil}}$, and $a_{0}$, which were derived from MRA1 for the measured attenuation spectra, without the MCS. Therefore, the values of b obtained from these 1800 sets of vectors a and concentrations $C_{m}$, $C_{\mathrm{oh}}$, $C_{\mathrm{dh}}$, and $C_{\mathrm{bil}}$ generated by the MCSs were used each time to estimate the concentrations from the measured spectra.

As the preliminary study, we performed *in silico* experiments with the diffuse reflectance spectra derived from the MCS to validate the proposed approach and to confirm the relationship between the regression coefficients and the values of $C_{m}$, $C_{\mathrm{oh}}$, $C_{\mathrm{dh}}$, and $C_{\mathrm{bil}}$. These spectra are considered as pseudo measured spectra. In this case, the reflectance spectra used in the main process were different from those used in the preparation work. In this MCS for *in silico* experiments, the absorption coefficients of the epidermis for 10 different lower concentrations of $C_{m}=1\%$ to 10% at intervals of 1% were input for the epidermis. The absorption coefficient of total hemoglobin $\mu_{a,\mathrm{th}}(\lambda )$ and the values for $C_{\mathrm{th}}=0.2$, 0.4, 0.6, 0.8, and 1.0 vol.% were used as input for the dermis layer in the MCS. Tissue oxygen saturation was assumed to be ${\mathrm{StO}}_{2}=60\%$ for all combinations. The values of $\mu_{a,\mathrm{bil}}(\lambda )$ for $C_{\mathrm{bil}}=0$, 1, 5, 10, 15, and 20  mg/dl were used as input for the dermis layer in the MCS. The reduced scattering coefficient $\mu_{s^{\prime }}(\lambda )$^38^ was used for both the epidermis and dermis. The refractive index of the epidermis and dermis layers was assumed to be the same and fixed at 1.4 for all simulations. In total, 300 diffuse reflectance spectra at λ=460 to 590 nm with a 10 nm interval were simulated under the various combinations of $C_{m}$, $C_{\mathrm{oh}}$, $C_{\mathrm{dh}}$, and $C_{\mathrm{bil}}$ described above.

**Fig. 2:**
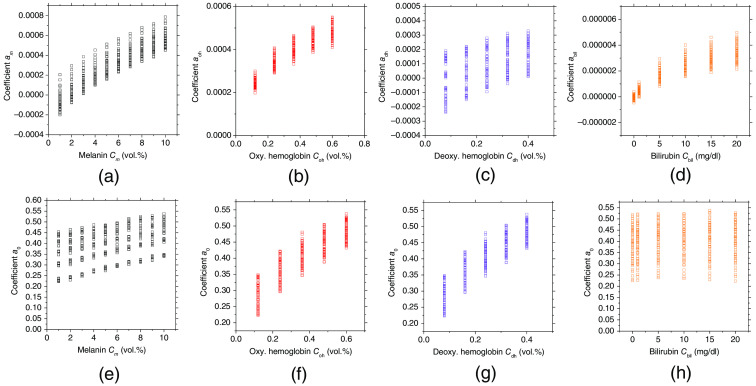
Regression coefficients versus concentrations of melanin, oxygenated hemoglobin, deoxygenated hemoglobin, and bilirubin obtained from the MCSs. (a) $a_{m}$ versus $C_{m}$ under the different conditions of $C_{\mathrm{oh}}$, $C_{\mathrm{dh}}$, and $C_{\mathrm{bil}}$. (b) $a_{\mathrm{oh}}$ versus $C_{\mathrm{oh}}$ under the different conditions of $C_{m}$, $C_{\mathrm{dh}}$, and $C_{\mathrm{bil}}$. (c) $a_{\mathrm{dh}}$ versus $C_{\mathrm{dh}}$ under the different conditions of $C_{m}$, $C_{\mathrm{oh}}$, and $C_{\mathrm{bil}}$. (d) $a_{\mathrm{bil}}$ versus $C_{\mathrm{bil}}$ under the different conditions of $C_{m}$, $C_{\mathrm{oh}}$, and $C_{\mathrm{dh}}$. (e) $\alpha_{0}$ versus $C_{m}$ under the different conditions of $C_{\mathrm{oh}}$, $C_{\mathrm{dh}}$, and $C_{\mathrm{bil}}.$ (f) $\alpha_{0}$ versus $C_{\mathrm{oh}}$ under the different conditions of $C_{m}$, $C_{\mathrm{dh}}$, and $C_{\mathrm{bil}}$. (g) $\alpha_{0}$ versus $C_{\mathrm{dh}}$ under the different conditions of $C_{m}$, $C_{\mathrm{oh}}$, and $C_{\mathrm{bil}}$. (h) $\alpha_{0}$ versus $C_{\mathrm{bil}}$ under the different conditions of $C_{m}$, $C_{\mathrm{oh}}$, and $C_{\mathrm{dh}}$.

Figure 2 shows the dependence of the regression coefficients obtained from MRA1 on the concentrations of the chromophores. Figure 2(a) shows the value of $a_{m}$ versus the volume concentration of melanin for various values of $C_{\mathrm{oh}}$, $C_{\mathrm{dh}}$, and $C_{\mathrm{bil}}$. Figure 2(b) shows the value of $a_{\mathrm{oh}}$ versus the volume concentration of oxygenated hemoglobin for various values of $C_{m}$, $C_{\mathrm{dh}}$, and $C_{\mathrm{bil}}$. Figure 2(c) shows the value of $a_{\mathrm{dh}}$ versus the volume concentration of deoxygenated hemoglobin for various values of $C_{m}$, $C_{\mathrm{oh}}$, and $C_{\mathrm{bil}}$. Figure 2(d) shows the value of $a_{\mathrm{bil}}$ versus the volume concentration of bilirubin for various values of $C_{m}$, $C_{\mathrm{oh}}$, and $C_{\mathrm{dh}}$.

In Fig. 2(a), the value of $a_{m}$ increases with the increase of $C_{m}$. Moreover, the value of $a_{m}$ changes with the increase in the values of $C_{\mathrm{oh}}$, $C_{\mathrm{dh}}$, and $C_{\mathrm{bil}}$. The same tendency can be seen for $C_{\mathrm{oh}}$, $C_{\mathrm{dh}}$, and $C_{\mathrm{bil}}$, as shown in Figs. 2(b)–2(d), respectively. There is not a simple linear relationship between regression coefficients and concentrations. This is due to the contribution of mean path length to the regression coefficient in addition to concentration. Each regression coefficient depends on all chromophore concentrations. This implies that the regression coefficient cannot simply be separated into C and l. This is the reason why we introduce MRA2 for the estimation of C. Figures 2(e)–2(h) show the values of $a_{0}$ versus the values of $C_{m}$, $C_{\mathrm{oh}}$, $C_{\mathrm{dh}}$, and $C_{\mathrm{bil}}$, respectively. The value of $a_{0}$ increases with the increase in the value of $C_{m}$. Moreover, the value of $a_{0}$ increases with the increases in $C_{\mathrm{oh}}$ and $C_{\mathrm{dh}}$. On the other hand, the value of $a_{0}$ is almost constant with an increase in the value of $C_{\mathrm{bil}}$. The different contributions of $a_{0}$ among all the C values can be explained by Eq. (8). In this way, the regression coefficients $a_{m}$, $a_{\mathrm{oh}}$, $a_{\mathrm{dh}}$, $a_{\mathrm{bil}}$, and $a_{0}$ are related to each chromophore concentration. For this reason, we use the regression coefficients to estimate the chromophore concentrations. However, $C_{m}$, $C_{\mathrm{oh}}$, $C_{\mathrm{dh}}$, and $C_{\mathrm{bil}}$ are not determined by a unique regression coefficient when using only MRA1. In other words, the regression coefficients $a_{i}$ cannot simply be separated into $C_{i}$ and $l_{i}$. Thus, we introduce the empirical formulae of Eqs. (9)–(12) derived from MRA2 to determine $C_{m}$, $C_{\mathrm{oh}}$, $C_{\mathrm{dh}}$, and $C_{\mathrm{bil}}$ using the regression coefficients of $a_{m}$, $a_{\mathrm{oh}}$, $a_{\mathrm{dh}}$, $a_{\mathrm{bil}}$, and $a_{0}$.

**Fig. 3:**
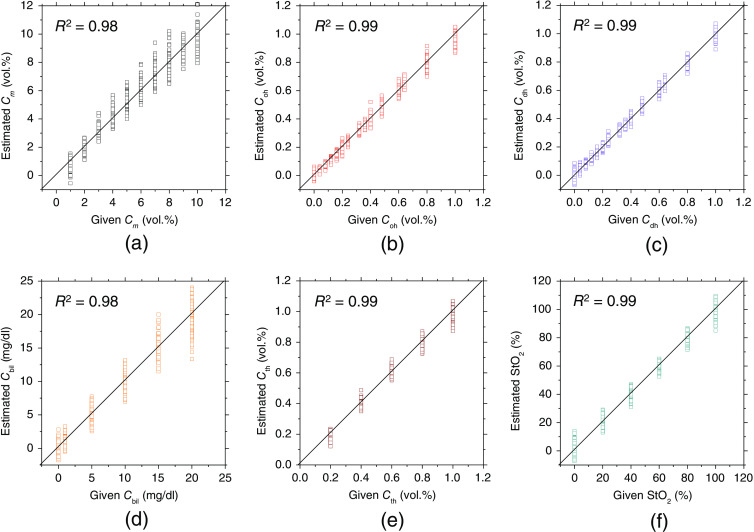
Comparisons between the estimated and given values for (a) $C_{m}$, (b) $C_{\mathrm{oh}}$, (c) $C_{\mathrm{dh}}$, (d) $C_{\mathrm{bil}}$, (e) $C_{\mathrm{th}}$, and (f) ${\mathrm{StO}}_{2}$, obtained from the numerical simulations.

Figure 3 shows the comparison between the estimated and given values for (a) $C_{m}$, (b) $C_{\mathrm{oh}}$, (c) $C_{\mathrm{dh}}$, (d) $C_{\mathrm{bil}}$, (e) $C_{\mathrm{th}}$, and (f) ${\mathrm{StO}}_{2}$. The multiple dots per column in Fig. 3 represent different conditions for the other chromophores. The words “given value” is the value of the chromophore concentration set in the MCS, that is, the ground truth value. On the other hand, the word “estimated” is the estimated value obtained by the proposed method. The estimated values correlate well with the given ones, indicating the effectiveness of the empirical formulae derived from MRA2.

## 3 Experiments

### 3.1 Measuring System

#### 3.3.1 Diffuse reflectance spectroscopic system

**Fig. 4:**
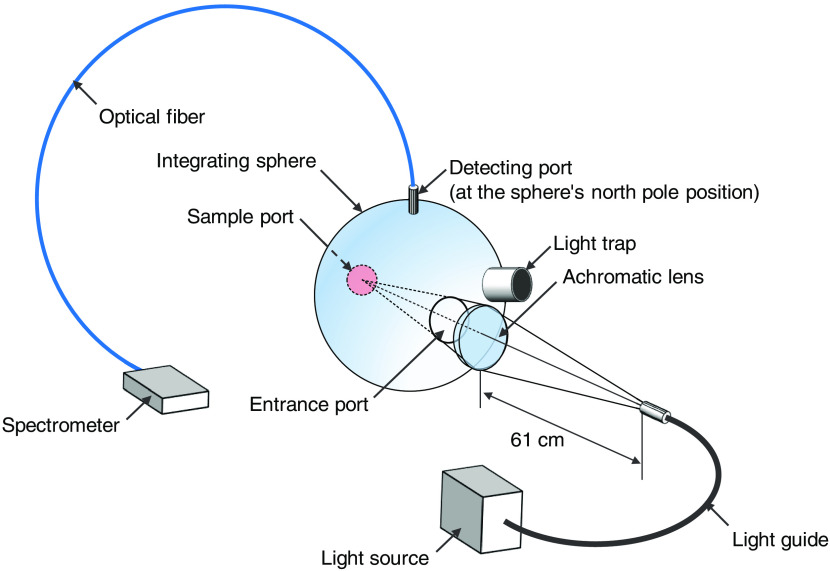
Schematic illustration of experimental setup for measuring diffuse reflectance spectra of rat dorsal skin.

Figure 4 shows a schematic illustration of the system for measuring diffuse reflectance spectra. A halogen lamp light source (LA-150SAE, Hayashi Watch Works Co., Ltd., Tokyo, Japan) illuminates the sample via a light guide and an achromatic lens with a spot diameter of 4.0 mm. The sample is placed at the sample port of an integrating sphere (RT-060-SF, Labsphere Inc., North Sutton, New Hampshire, United States). The detected area of the sample is circular, with a diameter of 2.2 cm. Light diffusely reflected from this area is integrated by the sphere and a portion of the diffuse reflected light is received at the input face of an optical fiber probe having a diameter of 400  μm placed at the detector port of the sphere. The detector port is located at the north pole of the integrating sphere while the sample port is located at the equator. This port arrangement is used in most integrating spheres. The fiber transmits the received light into a multichannel spectrometer (USB2000, Ocean Optics Inc., Dunedin, Florida, United States), which measures reflectance spectra in the visible wavelength range under the control of a personal computer (PC). A standard white diffuser with 99% reflectance (SRS-99-020, Labsphere Incorporated, New Hampshire, United States) is used to measure the reference spectrum $i_{\mathrm{std}}(\lambda )$ for calculating r(λ). For the measurement of a diffuse reflectance spectrum r(λ) with the integrating sphere, $i_{\mathrm{std}}(\lambda )$ is a reflected light spectrum taken with the standard white diffuser illuminated with the light from the light source while $i_{t}(\lambda )$ is a reflected light spectrum taken with the rat dorsal skin. The standard white diffuser was placed at the sample port of the integrating sphere. After measuring $i_{t}(\lambda )$, the standard white diffuser was replaced by the rat dorsal skin to measure $i_{\mathrm{std}}(\lambda )$. In the *in vivo* optical measurements, a single reflectance spectrum is obtained by averaging ten successive recordings of the reflectance spectrum, in which one recording is made with the integration time of 200 ms. Therefore, the acquisition of a single reflectance spectrum requires a total of 2 s.

#### 3.1.2 Diffuse reflectance imaging system

**Fig. 5:**
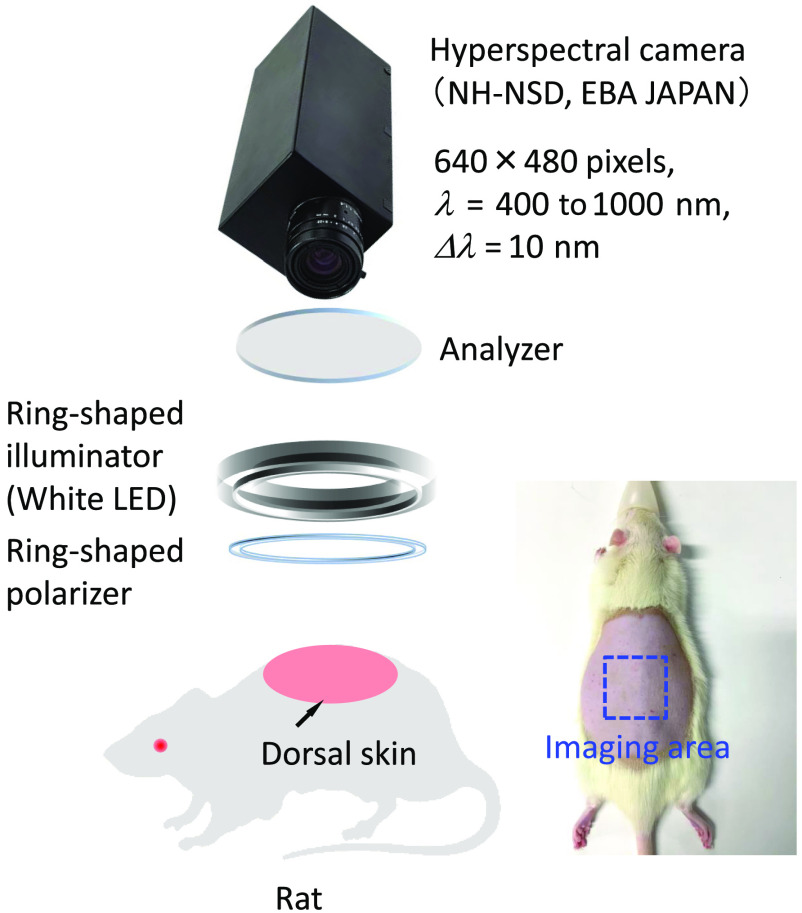
Schematic illustration of the hyperspectral diffuse reflectance imaging system.

Figure 5 shows a schematic illustration of the hyperspectral imaging system used in this study. A halogen lamp light source (LA-150SAE, Hayashi Watch Works Co., Ltd., Tokyo, Japan) illuminates the surface of a sample via a light guide with a ring-shaped illuminator. Diffusely reflected light is received by a hyperspectral camera (NH-NSD, EBA JAPAN, Japan) with a camera lens to acquire a hyperspectral cube. The hyperspectral cube consists of two spatial dimensions and a spectral dimension, in which the first two dimensions are spatial (x and y axes) with 640×480  pixels while the third dimension (z axis) is the wavelength, ranging from 400 to 1000 nm with a 10 nm interval. The standard white diffuser is used to measure the reference spectrum $i_{\mathrm{std}}(\lambda )$ for calculating r(λ). A ring-shaped polarizer and an analyzer are set in a crossed Nicols alignment to reduce specular reflection from the skin surface. The hyperspectral image data are then stored in a PC and analyzed according to the visualizing process described above.

### 3.2 Animal Experimental Protocols

Male Wister rats (n=12) and Male Long-Evans rats (n=2) weighing from 300 to 630 g were used for the animal experiments. All experimental procedures were conducted according to the protocols approved by the Animal Care Committee of Tokyo University of Agriculture and Technology (Approval numbers 22-28 and 31-25). Anesthesia of rats was performed with isoflurane and maintained at a depth such that the rat had no response to toe pinching. After induction of anesthesia, the dorsal region was shaved and a depilatory agent, including thioglycolic acid, was applied on the rat dorsal skin.

First, we performed spectral diffuse reflectance measurements and imaging of rat dorsal skin with obstructive jaundice. Obstructive jaundice was induced in seven Wister rats after common bile duct ligation. In the laparotomy, the ligamentous attachments from the liver to the diaphragm and abdominal wall were dissected to mobilize the liver lobes. The bile duct was exposed and carefully separated from the portal vein and hepatic artery. A suture was placed around the bile duct and secured with two surgical knots. The abdominal wall was closed with separate running sutures. Concurrently with the diffuse reflectance measurements, the transcutaneous bilirubin level was measured by a transcutaneous jaundice meter (JM-105 Konica Minolta, Inc, Tokyo, Japan).

Second, we carried out spectral diffuse reflectance measurements and imaging with five Wister rats while varying the ${\mathrm{FiO}}_{2}$. The value of ${\mathrm{FiO}}_{2}$ was regulated by mixing 95% $O_{2}$ to 5% ${\mathrm{CO}}_{2}$ gas and 95% $N_{2}$ to 5% ${\mathrm{CO}}_{2}$ gas in an arbitrary ratio. Hyperoxia (${\mathrm{FiO}}_{2}=95\%$) was induced by 95% $O_{2}$ to 5% ${\mathrm{CO}}_{2}$ gas inhalation, for which a breath mask was used under spontaneous respiration, whereas anoxia (${\mathrm{FiO}}_{2}=0\%$) was induced by 95% $N_{2}$ to 5% ${\mathrm{CO}}_{2}$ gas inhalation. To identify respiration arrest (RA), the respiration of the rat was confirmed by observing the periodical movement of the lateral region of the abdomen during the experiments. In the time series measurements of *in vivo* reflectance spectra while varying ${\mathrm{FiO}}_{2}$, single reflectance spectra were acquired at 10 s intervals for 40 min. The values of $C_{m}$, $C_{\mathrm{oh}}$, $C_{\mathrm{dh}}$, $C_{\mathrm{th}}$, ${\mathrm{StO}}_{2}$, and $C_{\mathrm{bil}}$ were calculated according to the estimation procedure for chromophore concentrations described above. Concurrently with the diffuse reflectance measurements, the percutaneous arterial oxygen saturation ${\mathrm{SpO}}_{2}$ was measured by a pulse oximeter (MOUSEOX Pulse Oximeter; Star Life Science, Oakmont, Pennsylvania, United States) to evaluate qualitatively the change in ${\mathrm{StO}}_{2}$ obtained by the proposed method.

Third, we performed spectral diffuse reflectance measurements and imaging of dorsal skin after hair removal with two Long-Evans rats. The dorsal hair of each rat was shaved by hair clippers and removed by applying a depilatory agent until the skin surface appeared. The dorsal skin of each rat was observed and photographed every 1 or 2 days following depilation.

## 4 Results and Discussion

### 4.1 Measurements and Imaging with Wister Rats During Obstructive Jaundice

**Fig. 6:**
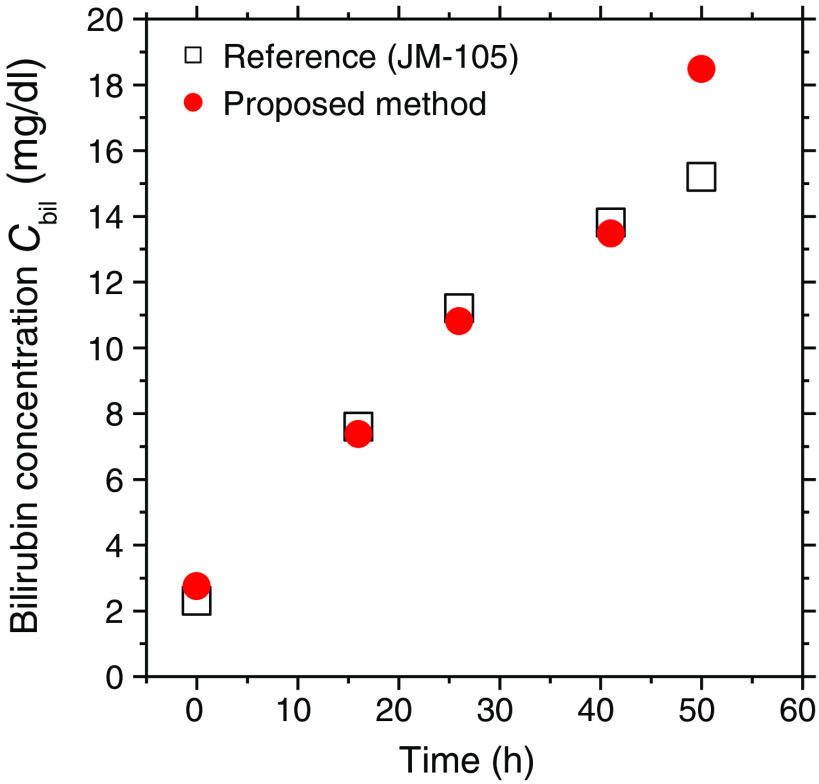
Typical time courses of transcutaneous bilirubin concentration $C_{\mathrm{bil}}$ estimated from the diffuse reflectance spectra measured by the spectrometer and those measured by a commercially available bilirubinometer before and after bile duct ligation.

Figure 6 shows typical time courses of transcutaneous bilirubin concentration $C_{\mathrm{bil}}$ estimated from the diffuse reflectance spectra measured by the spectrometer and that measured by a commercially available bilirubinometer before and after bile duct ligation.

**Fig. 7:**
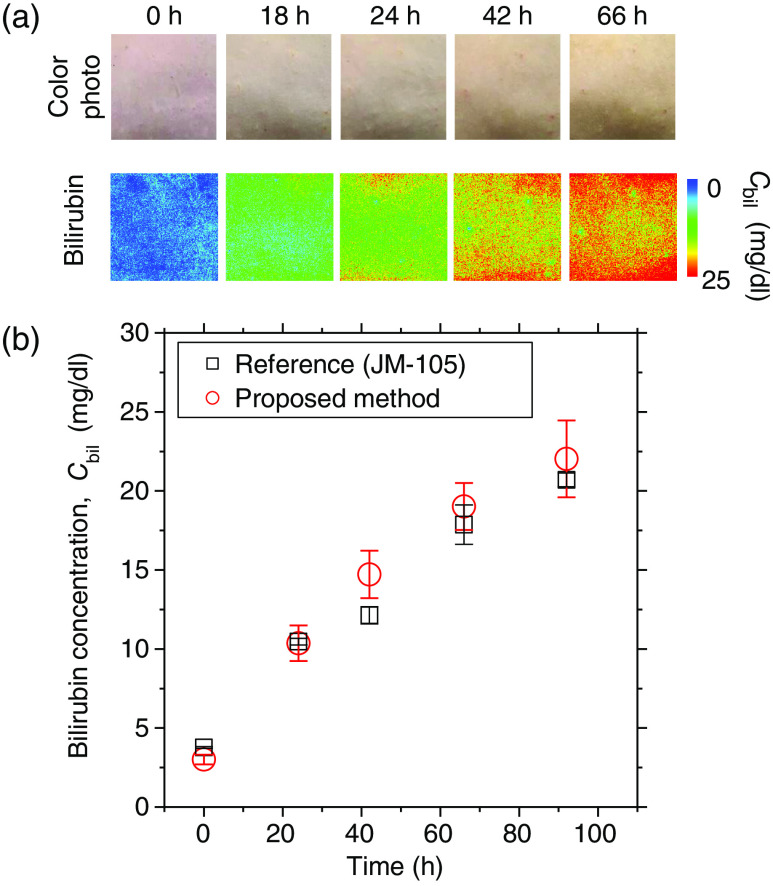
(a) Typical sequential images of $C_{\mathrm{bil}}$ obtained from the spectral diffuse reflectance images acquired by the hyperspectral camera before and after bile duct ligation. (b) The average value of $C_{\mathrm{bil}}$ over the entire region of the images shown in panel (a).

The estimated value of $C_{\mathrm{bil}}$ increases monotonically and reaches 18.5  mg/dl at 50 h after the onset of bile duct ligation, which shows the same tendency as the transcutaneous bilirubin concentration measured by the bilirubinometer. Figure 7(a) shows typical sequential images of $C_{\mathrm{bil}}$ obtained from the spectral diffuse reflectance images acquired by the hyperspectral camera before and after bile duct ligation. The value of $C_{\mathrm{bil}}$ averaged over the entire region of the images shown in Fig. 7(a) increased monotonically and reached 18  mg/dl at 66 h after the onset of bile duct ligation, which shows the same tendency as the results shown in Fig. 6.

**Fig. 8:**
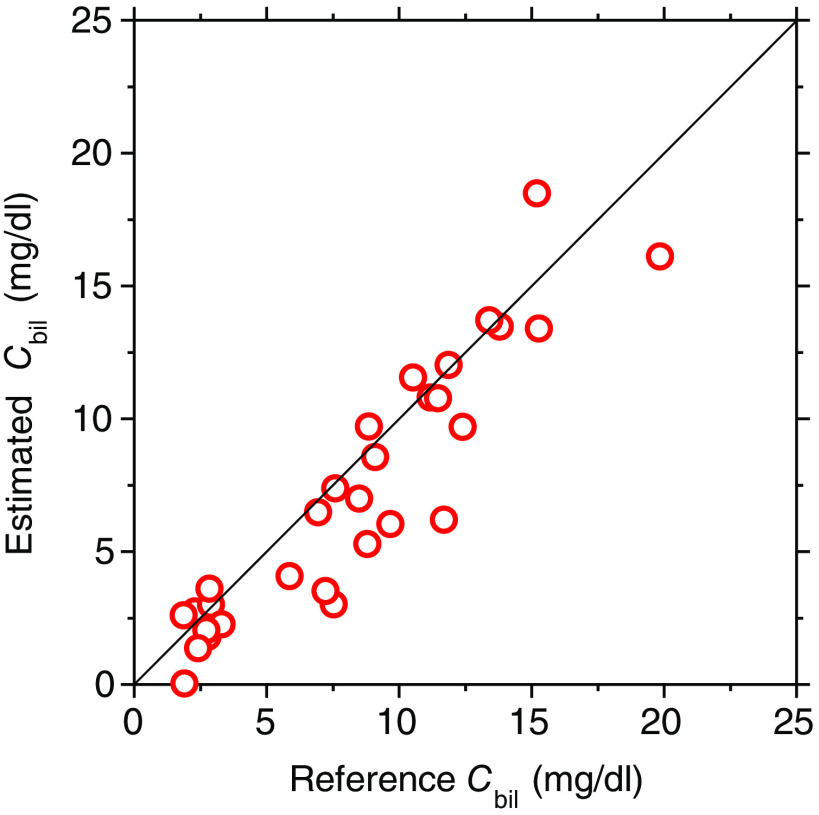
Comparison between the bilirubin concentration $C_{\mathrm{bil}}$ estimated by the proposed method and that measured by the commercially available bilirubinometer, obtained from seven samples during bile duct ligation.

Figure 8 shows a comparison between the bilirubin concentration $C_{\mathrm{bil}}$ estimated by the proposed method and that measured by the commercially available bilirubinometer, obtained from seven samples before and after bile duct ligation. The value of $C_{\mathrm{bil}}$ estimated by the proposed method agrees well with that measured by the transcutaneous bilirubinometer. The correlation coefficient between the estimated value of $C_{\mathrm{bil}}$ and the ground truth value is R=0.92 (p<0.0001).

**Fig. 9:**
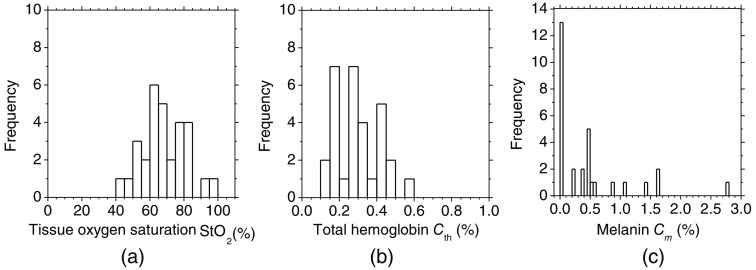
Histograms of estimated values for (a) the tissue oxygen saturation ${\mathrm{StO}}_{2}$, (b) the concentration of total hemoglobin $C_{\mathrm{th}}$, and (c) the concentration of melanin $C_{m}$, obtained from seven samples during bile duct ligation.

Figure 9 shows the histograms of (a) tissue oxygen saturation ${\mathrm{StO}}_{2}$, (b) total hemoglobin concentration $C_{\mathrm{th}}$, and (c) melanin concentration $C_{m}$ estimated by the proposed method, obtained from seven samples before and after bile duct ligation. The estimated values of ${\mathrm{StO}}_{2}$ were distributed within the range from 40% to 100%, having a single peak around 65%. The average value of 67.4%±12.5% for ${\mathrm{StO}}_{2}$ is lower than the typical arterial oxygen saturation ${\mathrm{SaO}}_{2}$, which ranges from 90% to 98%. The typical value of venous oxygen saturation ${\mathrm{SvO}}_{2}$ is around 60%. The value of ${\mathrm{StO}}_{2}$ estimated by the proposed method represents the oxygen saturation for the mixture of arterio-venous blood. Almost 75% of the total blood volume in the whole body is contained within the veins and venules,^42^^–^^44^ whereas 25% of it is contained within the arteries and arterioles. Assuming that the blood volume ratio of venules and arterioles in the skin tissue is similar to that of the whole body, and the value of ${\mathrm{SaO}}_{2}$ under the normal condition is 96%, the tissue oxygen saturation of skin is calculated to be 69%. This value is close to the average value of 67.4%±12.5% for ${\mathrm{StO}}_{2}$ obtained by our method. The estimated values of $C_{\mathrm{th}}$ were distributed within the range from 0.1 to 0.6 vol.%, corresponding to the typical cutaneous hemoglobin content reported in the literature.^17^^,^^32^^,^^38^ The results of $C_{m}$ exhibited very low values distributed within the range from 0 to 2 vol.%, which is consistent with the fact that albino rats were used in this study.

Delgado Atencio et al.^40^ have proposed a three-step inverse algorithm for the estimation of melanin, total blood, and bilirubin. In this algorithm, the first step is to estimate the concentration of melanin only on the basis of the diffuse reflectance at 700 nm. In the second step, only the blood concentration is estimated using the diffuse reflectance at 578 nm. In this step, the diffuse reflectance at 578 nm is generated by the MCS with the blood concentration as an input parameter, and an inverse function for the blood concentration is determined. In the final step, only the bilirubin concentration is estimated using the diffuse reflectance at 460 nm. In this step, the diffuse reflectance at 460 nm is generated by the MCS with the bilirubin concentration as an input parameter, and an inverse function for the bilirubin concentration is finally determined. This method requires three inverse functions to be generated for each measurement. In addition, it does not provide an estimate of the oxygen saturation of the blood. In comparison with that method, our method is novel in that it can estimate not only melanin, total blood, and bilirubin but also oxygen saturation. In addition, in our method, once the empirical formulas for each of the chromophore concentrations have been established, there is no need to generate them for each measurement. Thus, our method is simpler, faster, and more efficient than that proposed by Delgado Atencio et al.^40^

### 4.2 Measurements and Imaging with Wister Rats While Varying the Fraction of Inspired Oxygen

**Fig. 10:**
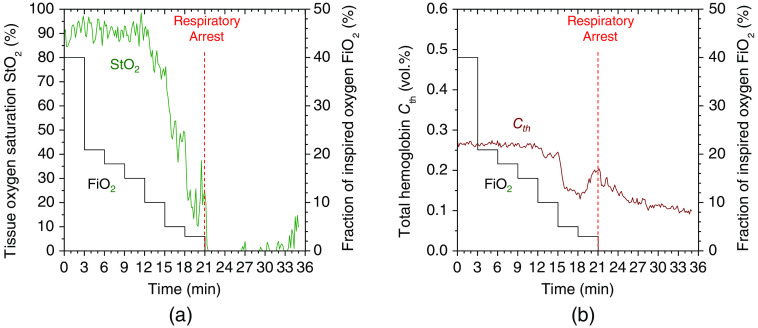
Typical time courses of (a) tissue oxygen saturation ${\mathrm{StO}}_{2}$ and (b) total hemoglobin concentration $C_{\mathrm{th}}$ obtained from the proposed method while changing ${\mathrm{FiO}}_{2}$.

**Fig. 11:**
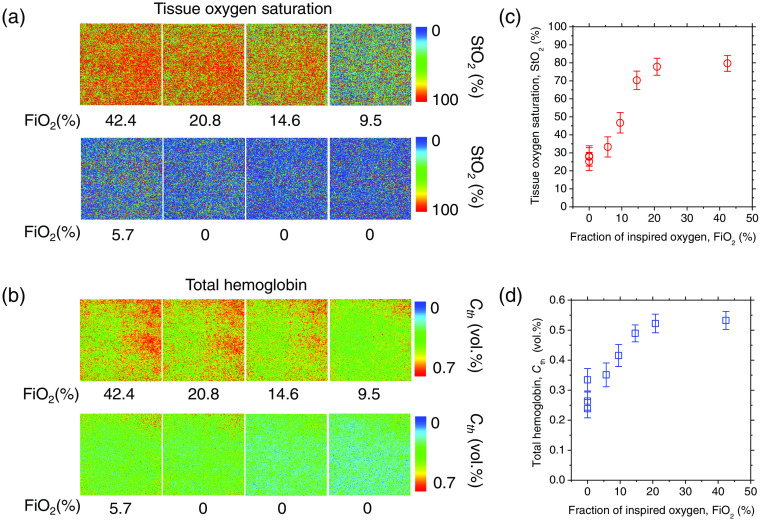
Typical sequential images of (a) tissue oxygen saturation ${\mathrm{StO}}_{2}$ and (b) total hemoglobin concentration $C_{\mathrm{th}}$ estimated from the spectral diffuse reflectance image acquired by the hyperspectral camera while changing ${\mathrm{FiO}}_{2}$. The average values of ${\mathrm{StO}}_{2}$ and $C_{\mathrm{th}}$ over the entire region of each image shown in (a) and (b) are plotted in (c) and (d), respectively.

Figure 10 shows typical time courses of (a) tissue oxygen saturation ${\mathrm{StO}}_{2}$ and (b) total hemoglobin concentration $C_{\mathrm{th}}$ estimated from the diffuse reflectance spectra measured by the spectrometer while changing ${\mathrm{FiO}}_{2}$. Figure 11 shows typical sequential images of (a) tissue oxygen saturation ${\mathrm{StO}}_{2}$ and (b) total hemoglobin concentration $C_{\mathrm{th}}$ estimated from the spectral diffuse reflectance images acquired by the hyperspectral camera while changing ${\mathrm{FiO}}_{2}$. The average values over the entire images shown in Figs. 11(a) and 11(b) are plotted in Figs. 11(c) and 11(d), respectively. The value of ${\mathrm{StO}}_{2}$ dropped sharply when ${\mathrm{FiO}}_{2}$ was below 18%, indicating the onset of hypoxemia due to hypoxia, as shown in Figs. 10(a) and 11(a). In Figs. 10(b) and 11(b), the value of $C_{\mathrm{th}}$ decreased when ${\mathrm{FiO}}_{2}$ was below 18%. This decrease in $C_{\mathrm{th}}$ probably reflects decreased peripheral perfusion resulting from increased cerebral perfusion in response to hypoxia. Hypoxic conditions put more stress on the central nervous system than on the distal system.^45^ Cerebral blood flow is preferentially protected over peripheral blood flow when hypoxia is detected in the brain. As a result, the cerebral blood flow will increase while the peripheral blood flow (blood flow to the muscles and skin) will decrease. Jia et al.^46^ reported that when ${\mathrm{FiO}}_{2}$ was changed from 20% to 10%, peripheral blood flow in rats transiently decreased to 70% to 82% of normal within 5 min, which is consistent with the result shown in Fig. 10(b). The value of $C_{\mathrm{th}}$ increased gradually after the onset of hypoxia and reached a local maximum immediately before RA, implying an increase in blood flow compensating for hypoxia. Immediately following RA, the value of $C_{\mathrm{th}}$ decreased gradually and became lower than that at hypoxia and normoxia. Time courses of $C_{\mathrm{th}}$ and ${\mathrm{StO}}_{2}$ while changing ${\mathrm{FiO}}_{2}$ were consistent with well-known physiological responses to changes in ${\mathrm{FiO}}_{2}$. In this study, it was difficult to validate the absolute values of ${\mathrm{StO}}_{2}$ and $C_{\mathrm{th}}$ estimated by our proposed method using commercially available devices or other methods. Instead, the estimated value of ${\mathrm{StO}}_{2}$ was compared with ${\mathrm{SpO}}_{2}$ measured by a commercially available animal pulse oximeter, which confirmed a correlation between ${\mathrm{StO}}_{2}$ and ${\mathrm{SpO}}_{2}$ (results are not shown here). When rats were transferred from normoxia to hypoxia, both estimated ${\mathrm{StO}}_{2}$ and measured ${\mathrm{SpO}}_{2}$ decreased. When the rats were returned from hypoxia to normoxia, both the estimated ${\mathrm{StO}}_{2}$ and the measured ${\mathrm{SpO}}_{2}$ increased and returned to their normal levels. Obviously, the accuracy of the absolute value of ${\mathrm{StO}}_{2}$ cannot be validated by the correlation between ${\mathrm{StO}}_{2}$ and ${\mathrm{SpO}}_{2}$ alone. However, we believe that the correlation between ${\mathrm{StO}}_{2}$ and ${\mathrm{SpO}}_{2}$ can provide qualitative support for the validity of the relative changes in ${\mathrm{StO}}_{2}$.

### 4.3 Measurements and Imaging of Dorsal Skin After Hair Removal with Long-Evans Rats

**Fig. 12:**
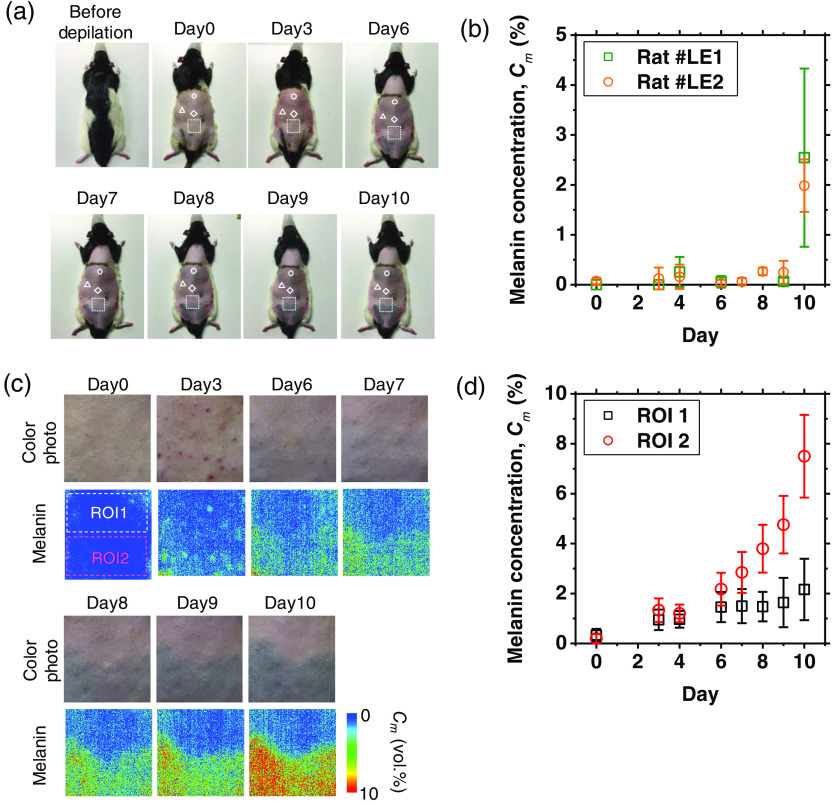
(a) Sequential photographs of the dorsal skin of a Long-Evans rat before and after depilation. Three symbols (a white open circle, a white open triangle, and a white open diamond) correspond to the three different spots measured by the spectrometer. A white dashed open square shows the imaging area of the hyperspectral camera. (b) Time courses of $C_{m}$ estimated from the diffuse reflectance spectra measured by the spectrometer after depilation for two rats. Each sequential plot is the average value over the two different spots. (c) Enlarged color photographs indicated by white squares shown in panel (a) and the corresponding image of $C_{m}$ estimated from the spectral diffuse reflectance image acquired by the hyperspectral camera. (d) Time courses of the average values over the ROIs on each image of $C_{m}$ shown in panel (c).

Figure 12(a) shows sequential photographs of the dorsal skin of a Long-Evans rat before and after depilation. The white open circle, white triangle, and white open diamond show the spots measured by the spectrometer. The white dashed square corresponds to the area imaged by the hyperspectral camera. The dorsal skin immediately after depilation appeared pale pink in color and turned gray gradually over time. Figure 12(b) shows the typical time courses of $C_{m}$ estimated from the diffuse reflectance spectra measured by the spectrometer after depilation for two rats. Each plot is the average value over the two different spots. The estimated values of $C_{m}$ started to increase at day 8 and reached 2 vol.% at day 10 after depilation.

Figure 12(c) shows enlarged color photographs indicated by white squares shown in Fig. 10(a) and the corresponding image of $C_{m}$ estimated from the spectral diffuse reflectance images acquired by the hyperspectral camera. The time courses of the average values over the regions of interest (ROIs) on each image of $C_{m}$ are also shown in Fig. 12(d). The estimated value of $C_{m}$ obtained from the ROI2 started to increase at 6 days after depilation and reached 7.5 vol.% at day 10, whereas that from the ROI1 increased more gradually. This is probably due to that they are in different stages of the hair cycle. One possible explanation for the sudden increase in $C_{m}$ on day 10 is likely because the total volume of all hairs per sample volume corresponding to ROI2 increased in a non-linear fashion over time, even though the hairs themselves grow in a linear fashion. Although there is a spatial variation in pigmentation, the temporal characteristic of $C_{m}$ shows a similar process as that plotted in Fig. 12(b).

The production of melanin is regulated by precise interactions in the hair follicle pigmentary unit involving follicular melanocytes, keratinocytes, and dermal papilla fibroblasts.^47^ The hair growth cycle consists of three distinct phases: growth phase (anagen), regression phase (catagen), and rest phase (telogen).^45^^,^^46^ The developmental steps for hair shafts consist of the production of melanin in follicular melanocytes, the transfer of melanin granules into cortical and medullary keratinocytes, and the formation of pigmented hair shafts. Hair pigmentation is active only during the growth phase (anagen stage) of the hair cycle, whereas the melanocytes in the skin continuously produce melanin. The production of melanin is inactivated in the transitional phase that allows the follicle to renew itself (catagen stage) and remains silent through the dormant phase (telogen stage).^47^ The production of melanin in follicular melanocytes during the anagen stage is connected with the regulatory system controlling hair growth such that a pigmented hair shaft is formed. The hair growth cycle in pigmented mice skin is associated with changes in skin color appearance^48^ because of the precise coupling of follicular melanogenesis and hair follicle cycling. The non-albino pigmented mice skin at the telogen stage appears pink in color and turns gray gradually during anagen development.^48^ Therefore, the dorsal skin with pale pink color immediately after depilation shown in Fig. 12(a) indicates that it was in the telogen stage of the hair growth cycle. The time course of $C_{m}$ shown in Figs. 12(b)–12(d) is indicative of the supply of melanosomes produced by melanocytes of the hair follicle to the growing hair shaft, which may be induced by depilation of skin on the back of Long-Evans rats that were in the telogen stage of the hair cycle.

## 5 Conclusions

In summary, a method for measuring and imaging bilirubin concentration, oxygenated hemoglobin concentration, deoxygenated hemoglobin concentration, and melanin concentration of skin tissues based on diffuse reflectance spectroscopy was demonstrated in the present study. We extended the method previously proposed^16^^,^^33^ to the quantification of oxygenated hemoglobin, deoxygenated hemoglobin, melanin, and bilirubin. We made it possible to analyze four chromophores in one method by newly introducing bilirubin absorption spectra into the predictor variables used in the MRA and the skin model for the MCS for light transport. *In vivo* experiments with rat dorsal skin after bile duct ligation showed good correlation in transcutaneous bilirubin concentrations between the proposed method and a commercially available transcutaneous bilirubinometer, which indicates the ability of the proposed method to evaluate transcutaneous bilirubin concentration. Time courses of total hemoglobin concentration and tissue oxygen saturation while changing the ${\mathrm{FiO}}_{2}$ coincide with well-known physiological responses to hyperoxia, normoxia, and anoxia, which demonstrate the feasibility of the method for monitoring skin hemodynamics due to loss of tissue viability and vitality. Although the studies to modulate oxygenation and bilirubin have been performed by other researchers, to evaluate the validity of our proposed method, comparison with known changes found in existing studies is important. Time courses of melanin concentration after depilation were consistent with follicular melanogenesis during the hair growth cycle, which shows the method’s possibility for evaluating melanogenesis in skin tissue *in vivo*. Evaluating skin melanin concentration by the proposed approach will not only be useful for monitoring follicular melanogenesis but also enable us to diagnose melanogenesis in several pigmented skin lesions and melanoma in the future. The results obtained by the long-term *in vivo* monitoring of melanogenesis in skin of rats are an addition to the existing literature.

## Data Availability

Data underlying the results presented in this paper are not publicly available at this time but may be obtained from the authors upon reasonable request and through a collaboration agreement.
